# Articulating a Patient-Centered Design Space for Cancer Journeys

**DOI:** 10.4108/eai.21-3-2017.152394

**Published:** 2017-03-21

**Authors:** M.L. Jacobs, J. Clawson, E.D. Mynatt

**Affiliations:** 1 College of Computing, Georgia Institute of Technology, Atlanta, GA 30308 USA

**Keywords:** Pervasive healthcare, patients’ needs, breast cancer, holistic care

## Abstract

Cancer journeys, encompassing patients’ cancer experiences through survivorship, are complex and diverse. Individuals must cope with numerous physical and emotional challenges, balancing clinical tasks alongside responsibilities of daily life. Understanding the breadth of factors that contribute to a patient’s cancer experience presents a critical challenge in developing holistic patient-centered technology. To further our understanding of the cancer journey, we conducted focus groups and interviews with 31 breast cancer patients. We present a cancer journey framework depicting the responsibilities, challenges, and personal impacts patients face while transitioning from diagnosis through post-treatment survivorship. Through this work, we aim to aid the development of health tools that consider a patient’s cancer journey and health needs more broadly, supporting patient’s health management alongside the complexities and priorities of daily life.

## 1. Introduction

For individuals battling cancer, the disease brings numerous physical, psychological, social, and personal consequences. Patients often must balance day to day responsibilities alongside new challenges such as severe fatigue and pain (1), a physical inability to work (2), and feelings of loneliness or isolation (3,4). While resources exist to help patients manage their healthcare, these tools focus on supporting a small subset of patient needs. An opportunity thus exists to develop technology that supports the broader cancer experience, helping patients to overcome both medical and personal challenges. Our work aims to inform the development of holistic health tools that broadly consider a patient’s cancer journey and health needs, to improve patient support and minimize the number of resources a patient must discover and incorporate into their healthcare management.

We present a holistic framework describing the cancer journey from the patient’s perspective. Using interviews and focus groups with 31 breast cancer patients, we developed the framework that reveals the range of responsibilities, challenges, and personal impacts patients must cope with while managing this disease. The framework highlights how each of these responsibilities, challenges, and personal factors change over the course of the cancer trajectory. Ultimately, we aim to provide guidance for technology designers and researchers developing tools to support the wide range of cancer patient needs. Through the framework we offer a detailed description of the cancer journey for those who wish to understand this complex design space but may not be able to conduct the longitudinal fieldwork necessary to capture patients’ experiences.

## 2. Related work

### 2.1. The cancer journey

Hundreds of cancer types exist, with over 14 million new cases of cancer diagnosed annually worldwide.^†^ Prior studies have identified common phases in cancer treatment that many patients encounter, including screening and diagnosis, information seeking, acute care and treatment, no evidence of disease, and chronic disease management (5). The physical and psychosocial symptoms patients experience change as they progress through various phases of care. For example, a person diagnosed with cancer is more likely to feel anxiety around the time of diagnosis. However, during treatments such as chemotherapy, anxiety may become less common and new symptoms emerge such as nausea and fatigue (6). In addition to physical symptoms, studies have provided greater understandings of patients’ health information needs (7–9), emotional coping strategies (10,11), and social support needs (3,12). Research has also conveyed the personal challenges that patients face, such as feelings of isolation and loss of self (4).

This body of work examining various aspects of patients’ cancer experiences reveals the complex make-up of cancer journeys. The challenge with understanding cancer journeys is that few papers discuss the full scope of patients’ experiences. Hayes et al. (5) provide one view of the cancer journey that motivated our earlier work. Their study reveals how cancer patients’ physical and emotional wellbeing change over time. This temporal account of the cancer journey inspired our interest in exploring how patients use technology throughout their cancer journeys (13). We found that participants discussed an even greater range of influences on their cancer experiences than covered by existing literature, leading to our analysis of the factors that make up one’s cancer journey. As a result, we present a description of the cancer journey that highlights the significant factors that contribute to one’s cancer experience. We expand on existing work by detailing the responsibilities, challenges, and personal effects that may occur during each phase of cancer care.

### 2.2. Tools to support cancer patients

A number of tools exist to help patients address personal health needs during cancer care. Such tools help patients to organize health information (14), manage their healthcare when away from home (15), reflect on their experiences (16), engage with their healthcare team (17,18), or foster social support (12). However, many existing health tools, while critical, support a subset of patients’ needs. Within the HCI community, designers of health tools have called for the need to incorporate more holistic support within modern patient health tools (19).

To date, little guidance is available to assist in the development of tools that consider both patients’ medical and personal needs. Moreover little advice exists for care providers regarding the diversity of patient needs and the potential of health technologies to address those needs. To address these gaps, we present a framework that articulates the many personal facets of patients’ cancer experiences. By consulting a holistic view of the patient-centered cancer journey, designers may consider how to develop tools to help patients cope with the numerous challenges that comprise their cancer experiences.

## 3. Method

### 3.1. Data collection

For the past three years we have worked closely with cancer patients and survivors to examine how holistic mobile health technologies may be used to support patients’ changing needs over time. In the process of working with our patients, we encountered a much broader range of factors that influence patients’ cancer experiences than currently represented in the literature. To identify patient needs, we ran interviews and focus groups with breast cancer survivors. Interview participants had enrolled in a study examining how patients used health technology to support their healthcare needs over time (13). Through these interviews, participants reflected on their cancer experiences, support needs, and the ways in which they used technology throughout the cancer journey.

**Figure 1:**
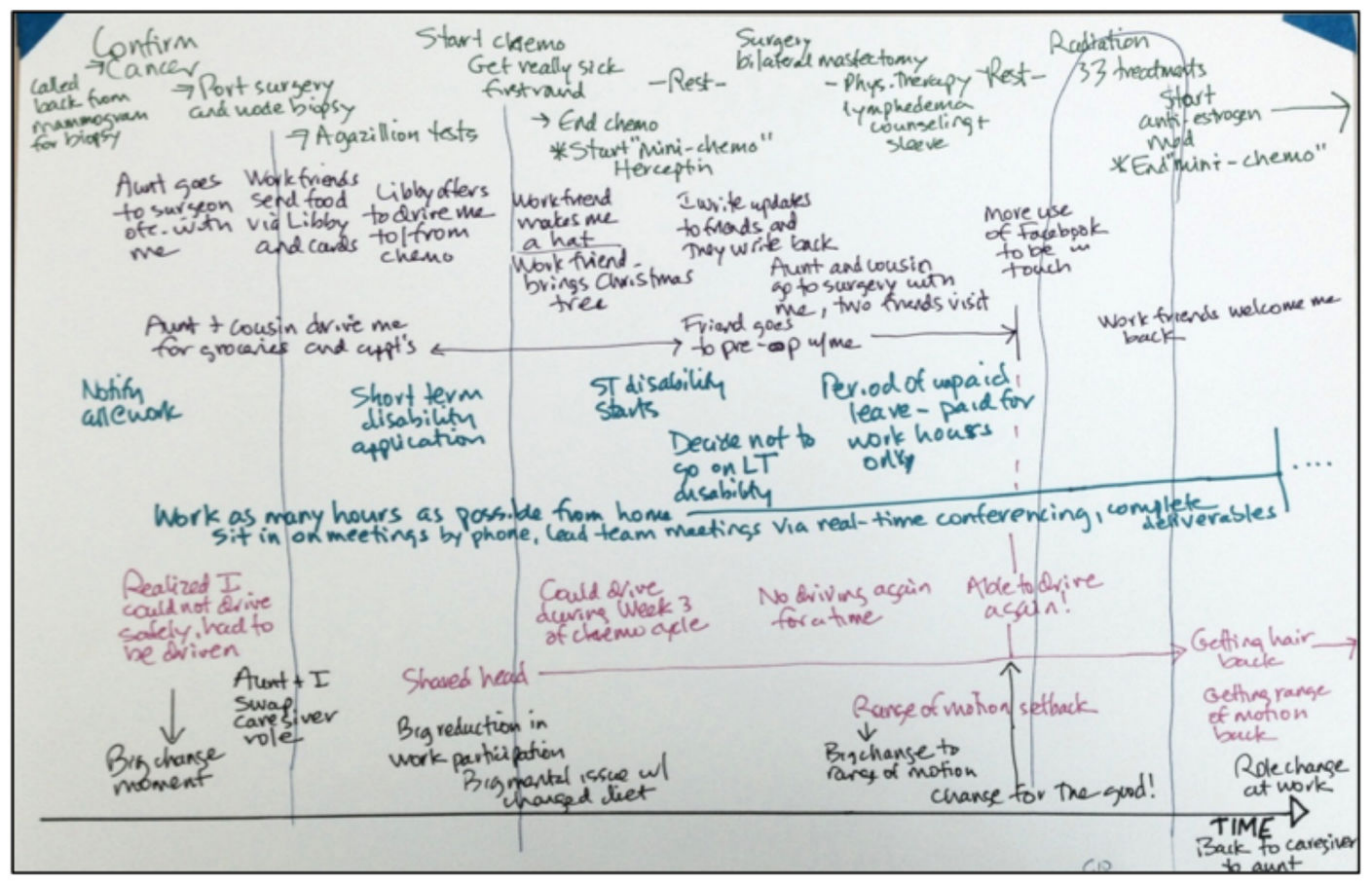
A sample cancer journey reconstruction

In a follow up study we ran focus groups to elicit discussions about the cancer journey in particular. Each focus group consisted of two to four participants. We asked participants to write down significant moments related to seven categories: medical; family and friends; work and finance; moments of change; problems or challenges; emotional highs and lows; and anything else that characterized their journey. We arrived at these categories based on our understanding of cancer navigator practices, as navigators also attempt to holistically address challenges throughout a patient’s cancer journey (20). We specifically asked participants to write or draw, as prior research has shown that this modality assists people in expressing their overarching mental models that are conceptually complex (21). By the end of the activity, each participant had developed a personal reconstruction of the cancer journey (figure 1). We recorded and transcribed all discussions.

In total, we interviewed 17 participants and conducted four focus groups with 14 participants. All 31 participants were going through or had recently completed cancer treatment, including surgery, radiation, and/or chemotherapy. The majority of interview participants were receiving cancer treatment at the time of the interviews. Focus group participants had completed active treatment and were beginning hormone therapy, with the exception of one participant who had not yet begun her chemotherapy treatment at the time of the focus group. All participants were recruited through and received treatment at the same cancer clinic in northwest Georgia. Participants’ ages ranged from 39–80, and all had been diagnosed with breast cancer stage 0–III.

### 3.2. Data analysis

The final dataset consisted of 14 cancer journey diagrams and 29 hours of transcription. In total, our dataset included 1,126 quotes that described personally significant moments that affected patients’ cancer experiences. Once we aggregated the data, five researchers individually reviewed the data and independently engaged in an open coding exercise. All researchers then compared codes and collaboratively developed an initial codebook. Through a subsequent iterative inductive analysis, we developed a finalized set of codes, organized into the high-level themes included in the cancer journey framework.

We organized our findings across four overarching cancer phases used in related literature (5): screening and diagnosis, information seeking, acute care and treatment, and no evidence of disease. While not all patients follow this treatment path, the phases help to demonstrate how patients’ needs and priorities shift over time, a critical component to consider when designing holistic health tools.

## 4. Findings

We organize our results into three categories: responsibilities, challenges, and how the cancer journey influenced patients’ daily life (their personal journey). The ‘responsibilities’ category highlights the multiple tasks that are placed on patients during each of the cancer journey phases. ‘Challenges’ includes specific issues participants encountered that served as barriers to receiving quality healthcare. Alongside these health-specific factors, all of our participants also grappled with dealing with cancer in the context of their daily lives. This part of the cancer journey can be unique to the individual, as each participant defined cancer in her own way. The ‘personal journey’ is patient-driven, changing based on our participants’ individual goals and needs.

**Figure 2:**
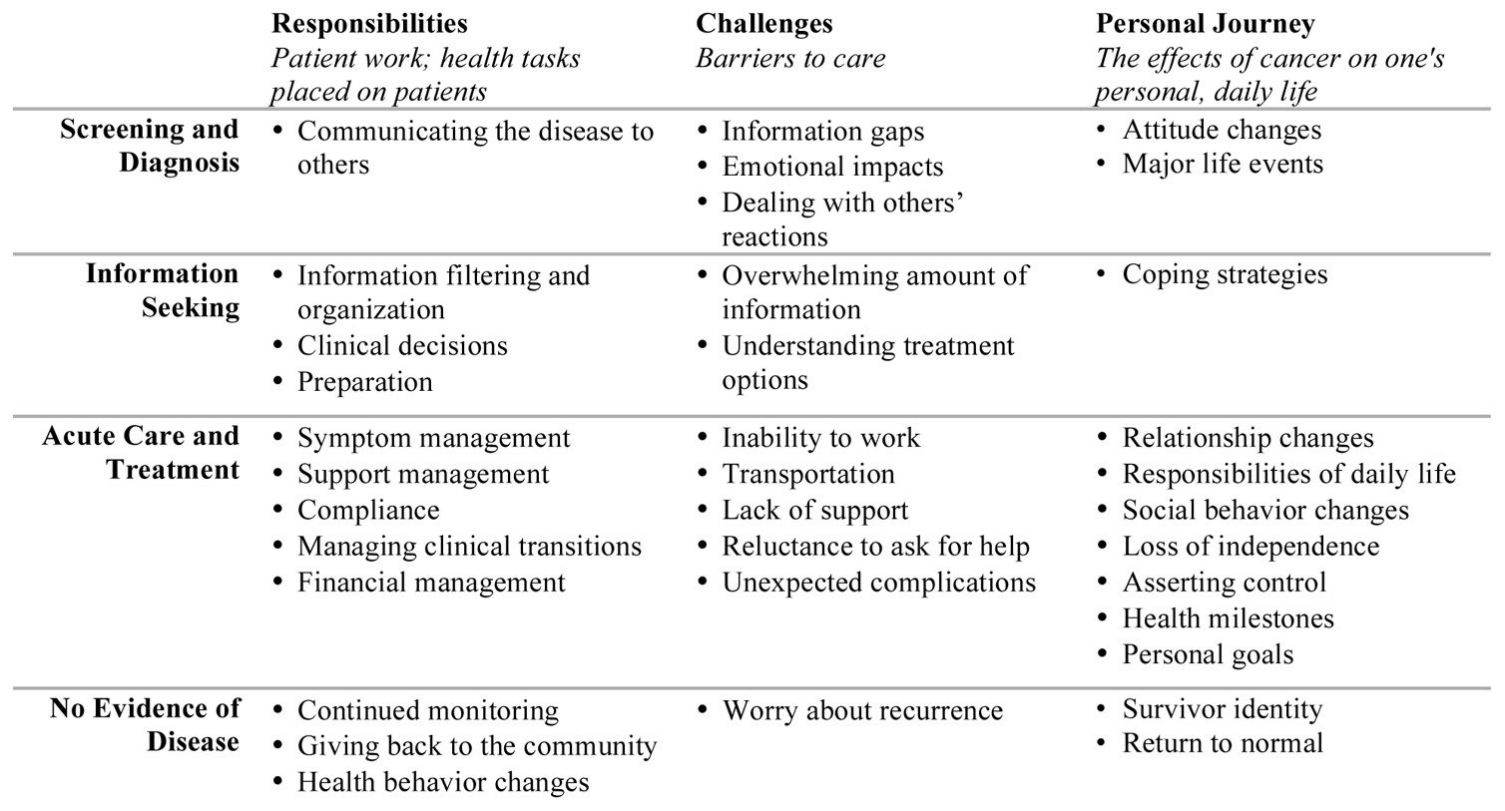
The cancer journey framework, representing the patient-centered cancer experience.

Figure 2 shows the cancer journey framework, depicting how patients’ responsibilities, challenges, and personal journey change over time. While we present each finding within the phase of the journey where participants most frequently described these themes, patients’ cancer experiences are personal and unique. Thus, the responsibilities, challenges, and personal factors may be present in several phases of the journey depending on an individual’s personal experiences.

### 4.1. Screening and diagnosis

This phase of the cancer journey typically begins when a patient experiences symptoms or participates in scheduled preventive screenings. Participants shared a number of responsibilities and challenges, showing the immediate effects a cancer diagnosis can have on one’s life.

#### Responsibilities

Upon diagnosis, 16 participants revealed they felt immediately responsible for **communicating the disease** to others. Telling children about the diagnosis was particularly difficult for our participants. Four participants recalled trying to stay positive during this particular discussion. Another four participants stated that telling children and grandchildren, while difficult, was a very important educational moment, as ensuring their family understood the diagnosis and family risks became a priority.

#### Challenges

One significant challenge 14 participants highlighted involved dealing with **information gaps**, or information they wish they had possessed. The most common gaps included understanding whether side effects were normal, the physical implications of treatment, and preventive cancer measures. At times, these information gaps can lead to critical issues. One participant had to deal with an unanticipated inability to work. Another participant took an unnecessary trip to the ER when she mistook a common side effect for a more serious issue. Our participants noted two differing causes of these information gaps. Three participants stated that they wished to have greater access to information about others’ similar experiences. However, another three participants stated that they felt they received too much information, making it *“hard to assimilate.”* Additionally, participants struggled to deal with conflicting information. One participant, for instance, shared a time in which she received different information from the multiple providers she was working with, stating, *“They sent me to the radiation oncologist across the road. And then they said ‘what are you doing here? You’re not even a candidate for it’. Go back to your doctor.”*

20 of the 31 participants described **emotional impacts** they dealt with while going through their cancer journey. Participants shared a range of emotions resulting from their cancer diagnoses, such as fear, anger, anxiety, uncertainty and loneliness. One participant highlighted the range of emotions she felt at diagnosis, saying, *“Being diagnosed with breast cancer in itself was a complete change because there were fret, doubt, uncertainty, emotions ran high.”* Four participants shared that they experienced depression upon diagnosis.

Eleven participants struggled with **others’ reactions** to the diagnosis. Five participants stated that seeing their families’ fear was particularly difficult. Further, three participants described awkward or unsupportive reactions from other people, such as sharing *“stories of lost loved ones who died from [cancer].”* Participants then had to consider not only how to cope with their diagnosis, but also how to handle others’ reactions as well. Thus, while support can be valuable and well intentioned, it occasionally can become more of a burden than a benefit to patients.

#### Personal Journey

Eight participants shared a number of positive **attitude changes** that resulted from the cancer diagnosis. Examples of these attitude changes include, *“living life to the fullest,” appreciating everything god has given me,” and “appreciating the here and now.”* Such attitude changes exemplify some of the positive outcomes that emerged from the negative health experiences.

Occasionally, **major life events,** unrelated to one’s cancer journey but occurring during the cancer experience, became integrated with one’s reflection of the journey. Fourteen participants discussed significant life events when sharing their cancer experiences. For example, seven participants had family members pass away just prior to or during their cancer treatment. Five participants discussed close friends or family members who had recently been diagnosed with a serious illness. An additional three participants shared that they had lost their job within the months leading up to their cancer diagnosis. Participants discussed these life events and their cancer experiences interchangeably. While these life events may occur at any of the cancer journey phases, patients must figure out how to manage these events alongside their cancer management on a daily basis. The presence of these events reveals that one’s personal health management is not always her or his top priority. Thus, health tools must allow patients to effectively and efficiently manage their care without constantly requiring the prioritization of one’s cancer care.

### 4.2. Information seeking

The information-seeking phase describes the time between diagnosis and beginning treatment. During this time, patients often focus on aggressively searching for information about the disease and treatments.

#### Responsibilities

Participants often discussed the need to find and **filter health information** to help them better understand their particular diagnoses and treatment plans. While all participants looked for medical information about cancer, several participants also cautioned against looking online. Some participants recommended limiting Internet searches to only sites recommended by doctors. Thus, patients are responsible for not only finding medical information but filtering the information as well.

All of our participants also needed to make important **clinical decisions** regarding their treatment. Patients must make a number of significant decisions, choosing between various surgeries (mastectomy, lumpectomy), treatments (chemotherapy, radiation), and selecting doctors. Each decision has considerable consequences. Several participants shared stories similar to one participant who said, *“Having radiation treatment is not an easy thing to do, or a fun thing to do. And there are consequences even though it sounds kind of benign in its own way. So I decided to have a mastectomy [without radiation].”*

Fourteen participants also talked about the need to **prepare** for upcoming treatments. Participants discussed a variety of preparation tasks. Most participants would read about upcoming treatments and organize their calendar with appointment information. A few participants also wrote down questions to ask doctors prior to appointments, and talked to survivors who completed similar treatments about their experiences.

#### Challenges

In this phase of the journey, patients must independently manage an **overwhelming amount of information**, often causing additional stress. Nearly all of our participants discussed feeling overwhelmed due to the amount of information presented, and feeling obligated to go through it all. One participant stated, *“They told me so much that my head just buzzes now.”*

Feeling overwhelmed can be an especially critical challenge; as such feelings at times caused participants to disengage with their healthcare entirely. As one participant stated, when feeling overloaded *“your whole positive attitude just crumbles.”* Three participants highlighted that this stress, and the limited time between diagnosis and treatment, made it difficult to **understand their treatment options**. These participants stated that they wished they had more time to process this information, which was often provided to them in large cancer binders.

#### Personal Journey

As participants moved forward in their cancer care, the effects on their daily life became more prominent, forcing patients to directly confront how they would cope with this new health situation. To manage the range of emotions and mental changes, 16 participants discussed a various **coping strategies**. Seven participants focused on religion and prayer when they felt stressed. Another six participants emphasized the need to stay preoccupied through work, social events, or online games to stop them from dwelling on the diagnosis and future treatments. Two participants stated they only looked up information about the most immediate next step so they would not feel overwhelmed by the different treatments included in their care plans. One participant said that she would *“try to act like I don’t have it.”*

While individuals differed in how they handled the diagnosis, participants strived to find a successful coping mechanism.

### 4.3. Acute care and treatment

A number of treatments are used in breast cancer care, including chemotherapy and radiation. The types, dosages, and frequencies of treatment vary across patients. Nevertheless, commonalities exist as patients work to manage their treatments. This phase of care can be the most time intensive and physically demanding.

#### Responsibilities

**Symptom tracking** becomes critical during this phase of care so physicians understand how patients are reacting to their treatments (14). Participants discussed a range of physical side effects such as pain, bruising, nausea, losing fingernails and hair, and fatigue. Participants frequently discussed the need to actively manage side effects by keeping nurses informed.

Patients found emotional and social support came from many different sources. Unsurprisingly, the majority of our participants discussed that their greatest support came from close friends and family. Participants shared numerous stories in which the support exceeded their own expectations. For example, one participant stated, *“One friend told me that she and her husband were with me for “the long haul”. And they have been!”* Some participants also found support through their healthcare providers. As one participant shared, *“The doors open and it’s the nurse from the breast center. She’s just been with me everywhere I turn. I felt like, well I don’t have to worry.”* Many of our participants also valued the support they received from other cancer survivors. Several participants recommended that future patients *“talk with others who have it.”*

The makeup of individuals’ support networks differed amongst our participants. For some, coworkers and “church families” represented their core network, while others relied more specifically on immediately family members. Despite this variability, **support management** became a necessity for all of our participants as they shared their health information and updates with their support network. Participants did this in a variety of ways. One participant shared that she delegated responsibilities, asking one person to drive her to and from chemo, and another to help specifically with domestic chores. Often, this management requires the patient to be both organized and proactive. As one participant recommended, *“Be proactive and set a schedule for help. Say that you want to be driven to the grocery store.”*

In addition, treatment **compliance** becomes one of the most important responsibilities placed on patients in order to ensure effective treatment.

21 participants highlighted the importance of **managing clinical transitions** as participants completed surgery, began various treatments, and transitioned into post-treatment survivorship. Many participants talked about feeling the most anxious during these transitions. Five participants in particular echoed sentiments that transitions were each a *“learning curve,”* marked by uncertainty.

**Financial management** was a significant task for 14 participants, with several stating it was the most difficult and stressful task they faced both during and after treatment. Four participants did not have health insurance to cover the costs of treatments, and five participants did not have a regular source of income. However, even for those participants with a job and insurance, financial management proved very difficult. As one participant stated, *“even with insurance, this year has been crippling having to pay the 20% that insurance didn’t cover.”* While some participants admitted that they chose to ignore the bills in order to emotionally cope with the financial burden, others stated that they dealt with the burden by looking for grants and other financial resources, or asking for help from family and friends. For example, one participant shared that her family members bought her groceries during active treatment so that she could pay her medical bills.

#### Challenges

Our participants discussed a range of challenges they faced while in treatment, highlighting the diverse needs that arise during this time. Ten participants became physically unable to work due to extreme fatigue and pain. An **inability to work** often led to financial stress and reduced social support.

**Transportation** became a challenge for seven of our participants, as many lost the ability to drive themselves. Participants marked this inability to drive as a particularly significant change, as they now depended on family and friends to attend treatments.

Social support, while valued by all of our participants, was not always available. Seven participants recalled moments when they **did not have adequate social support**. For instance, one participant shared that her worst moments during the cancer journey were *“times when you needed family and they were not there to support”*.

For nine participants, **a reluctance to ask for help** occurred in parallel with an increased need for help. For three participants, this reluctance stemmed from not wanting to feel like a burden to others. Another two participants shared that they felt as though they were lucky to have received a treatable diagnosis, and therefore did not wish to draw attention to themselves.

In addition to the expected side effects, thirteen of our participants also dealt with **unexpected health complications.** These complications included infections and allergies to medications. Occasionally these complications had serious impacts. For instance, one participant spent a week in the hospital after her blood sugar became dangerously low following a chemotherapy treatment. Another participant was forced to postpone her cancer treatment and stay in the hospital when her drainage tubes became infected after surgery.

#### Personal Journey

The consequences of cancer treatments on one’s daily life are numerous. Eight participants shared how **family relationships changed**. For two participants, their children moved closer in order to take care of them. In another situation, a participant was the caregiver for her elderly aunt, but said they swapped roles while she went through cancer treatment. Three participants stated that through the cancer experience they became closer with family members.

Participants also talked about many **responsibilities of daily life**, which had to be balanced alongside treatment. While not directly related to cancer, these parallel responsibilities became embedded in participants’ cancer experiences. For example, the participant who lost her husband just prior to her diagnosis stated, *“I was very busy dealing with the details following my husband’s death. As executor of his will, I had many responsibilities.”* Another participant shared that she continued her college studies throughout her diagnosis and treatment. In an extreme case, one of our participants shared all of the external stresses she was dealing with in addition to her cancer, sharing, *“My 88 year old father was in a car wreck, hospital and rehab for 6 to 8 weeks. Then my breast cancer. Then my son’s mother in law was a victim of home invasion. And then an uncle we had gone to see fell and broke his neck and died. And that happened in four months. Of all of those, breast cancer’s been the least to me.”* Thus, not only do patients gain a number of new responsibilities as they manage their disease, but they must also balance a variety of ongoing commitments and tasks, limiting the time and energy devoted to healthcare management.

Six participants discussed **social behavior changes**, such as a new focus completing their “*bucket lists,*” making new friends, volunteering, and participating in new social activities. These six participants highlighted the importance of participating in social events to maintain a positive attitude. As one participant, who joined a sports league after her diagnosis, summarized, *“The men in the softball league were sweethearts. The sport kept me busy, gave me purpose. You really need a purpose to live.”*

An increased need for help often contributed to participants’ feeling a **loss of independence**. Six participants shared that they relied on others for a range of tasks, including *“mental and financial support.”* These participants all described this realization as a significant moment of change that was difficult to accept. One way participants managed this loss of independence was by purposefully **asserting control** when possible. Six participants discussed ways in which they asserted their control during treatment. Four participants shared that they decided to make cancer care decisions independently, occasionally opposing doctor or family recommendations. Thus, patients need to not only feel supported but also empowered. We heard repeatedly that participants did not want to be defined or categorized as patients but to be treated as people. Celebrating independence while also helping people secure the support they need is a delicate challenge.

While many negative consequences can come from a cancer diagnosis, six participants celebrated positive life **milestones** that occurred during their cancer journey. Finishing treatments served as one commonly discussed health milestone, especially after subsequent tests came back with good results, which as one participant stated, *“produced euphoria.”*

A few participants also shared **personal goals** that they continued to work towards during and after cancer treatment. Many participants discussed wanting to learn to better manage their health and wellness and improve their overall quality of life. Others talked about more short-term goals, such as maintaining straight A’s in their coursework. While extremely variable, discussing these goals help to reveal insights into participants’ focus and priorities, and could thus be information used to better tailor support to the individual.

### 4.4. No evidence of disease

Upon completing treatment, patients with non-terminal cancers can reach a point in which no evidence of the disease remains in the body. While patients are considered cured at this phase, **continued testing** becomes critical to monitor for cancer recurrences. Continued treatments, such as hormone therapy may also be used during this phase.

#### Responsibilities

Several participants shared a desire to **give back to others** as a cancer survivor. Participants felt a need to volunteer or share their knowledge with others once they completed treatment. Nine participants also felt obligated to take better care of their physical wellbeing. **Health behavior changes** included going to their general physicians more, keeping up to date with health checkups, and being more vigilant with their diets. For one participant, these health behaviors extended to her family as well, as her daughter began scheduling regular mammograms.

#### Challenges

A well-documented challenge people face after a cancer diagnosis is the concern of a **recurrence** (22). Our participants also described this post-treatment anxiety frequently. Many of our participants expressed feeling this once their active treatment ended. As one participant stated, *“I think I will always have fear of a possible reoccurrence even though tests are positive.”*

#### Personal Journey

Thirteen participants shared different ways in which they embraced this new aspect of their lives after treatment, taking on different **cancer identities**. Three participants identified as cancer survivors, with one participant sharing that she is now *“a big spokesperson for mammograms and checkups.”* In contrast, five participants said they felt that cancer was just an obstacle to get through, stating, *“this is a bump in the road,” “I refused to be permanently labeled as a cancer survivor,” and “I never really classified myself as a cancer victim.”* Understanding these different attitudes toward cancer after treatment may help to provide tailored, ongoing support to survivors.

As participants completed treatment, resuming **normal** routines became another significant milestone. As the side effects wore off, ten participants shared their excitement about resuming routines such as *“eating again”* and *“being able to drive again”.* However, resuming normal routines did occasionally feel bittersweet. Two participants shared that they felt sad about no longer regularly seeing the doctors, nurses, and friends they made during treatment.

## 5. Discussion

Throughout this paper we have presented a framework to allow researchers, designers, and healthcare professionals to understand the diversity of factors that comprise a person’s cancer journey. The results of this work highlight the robust range of responsibilities, challenges, and personal factors that patients grapple with while managing their cancer care.

The need for holistic support arises not just from meeting patient needs, but perhaps just as importantly, focuses more broadly on the life of the individual as a whole, moving beyond a narrow focus of illness management. While a single application or technology may not practically be able to address patients’ wide variety of needs, a coordinated system of tools and resources may provide the necessary level of holistic and flexible support that patients need to successfully navigate their cancer journey. Thus, as technology designers, we must ensure that patients have access to tools that collectively support their range of needs. We developed the cancer journey framework to guide this pursuit. One practical method for leveraging this framework may be to aggregate informational resources pertaining to the responsibilities and challenges common in a particular phase of care. In the remainder of the paper we discuss how this framework contributes to, and highlights the importance of, developing holistic support.

### 5.1. Why holistic support matters

Throughout this paper we have argued for the need and utility of a comprehensive framework to understand the diversity of factors that comprise a person’s cancer journey. Our confidence in this perspective continued to grow throughout this research process.

Cancer invades a person’s life, not just their body. The dominant clinical point of view ingrained in health information technologies reflect an approach to cancer care that requires patients to become healthcare experts and, at best, surrounds the patient with a new cancer-focused network: a loosely coordinated set of disease-focused processes, people, and information. However, in reality, a person is already surrounded by a multitude of networks: employment, family, hobbies and more. A cancer diagnosis invades this constellation and exerts a strong influence on the individual and the networks in which they already belong. A need exists for cancer care to better support the whole patient experience, balancing the clinical needs alongside the complexities and priorities of daily life. Our articulation of a cancer journey is a critical step along that path. The data gathered in this research amplifies and strengthens this point of view and provides many examples in which one’s cancer care affects or is affected by one’s personal life.

For example, our framework calls attention to many obstacles that can derail successful treatment, thus highlighting the need for tools to help individuals **remove non-clinical barriers to care**. A salient, non-clinical example includes logistical management. As participants lost the ability to drive, accessing work, social events, and treatments became increasingly difficult. Our patients struggled to manage this need alongside their treatment regimens, yet no tools exist that help patients anticipate and plan logistical support as part of their cancer care. Patients reported they often grappled with anticipating their overall support such needs, communicating those needs, and orchestrating that support. Further, participants often offered advice for new patients on how to find and organize logistical support. Tools that use patients’ logistical information and recommend local resources, like cancer navigator programs, can meet patients “where they are” and work to lower barriers to effective care.

Our framework calls attention to **providing support across different phases of care**. Our patients struggled with preparing for treatments and side effects. They needed support that reached across different phases, allowing them to explore treatment options as well as prepare for treatments. Our participants also struggled with the support throughout the transitions in their cancer journeys. They emphasized the feelings of uncertainty that characterize these transitions. A constant base of support can provide comfort and continuity during stressful transitions. Developing platforms that curate and promote programs and applications that connect patients to both their personal networks and newly acquired cancer-focused networks could scaffold the support they need, when they need it. Patients need support for planning for the next phase of care albeit with tools that reflect their individual needs and priorities, not just tools limited to a clinical focus.

Most health IT systems focus solely on clinical care. In fact some reimbursement regulations in the US mandate that health tools NOT provide general capabilities that have a commercial market value^‡^. Our framework calls attention to the value of **open platforms** that can meet legitimate needs that occur alongside the entirety of the cancer care. One simple example is providing games, media and tools that reflect personal interests as these can serve as valuable distractions and support personal goals and milestones (13).

We heard repeatedly that the people in our study refused to be defined by their cancer diagnosis and many expressed specific strategies in reaffirming existing activities or finding new activities that reflected their true **identity**. A nuanced design challenge is how to create tools that recognize that cancer invades not just the body, but also many facets of daily life, without redefining all of life as a cancer journey. A possible, yet whimsical, opportunity is a master switch that “turns cancer off” from the computing device when patients need a break and reintegrates cancer information when desired. Such a switch could allow a patient to check their email and calendar without being inundated with cancer-related information.

The need for holistic support is driven both by the need to assist patients, and the opportunity to directly improve the care provided. Developing holistic support calls for the removal of barriers to care, providing assistance across phases of care, the use of open platforms, and reflecting an individual’s true identity. Our work helps lead the development of technology that contributes to this holistic care objective. In the remained of this section we provide examples and tools for using the framework to motivate the design and development of health systems.

### 5.2. Designing for a cancer care phase

Each row of the framework describes a common phase in cancer care, including diagnosis, information seeking, treatment, and no evidence of disease. By focusing on the details of a particular phase, we may use the framework to develop tools that support a range of responsibilities, challenges, and personal implications that result during this treatment phase. For example, during the acute care and treatment phase, participants discussed a number of social responsibilities and challenges. Participants were often required to manage their support network, felt a reluctance to ask for help, and experienced relationship changes. In parallel, many of our participants dealt with a number of logistical issues including symptom management, clinical transitions, and financial management.

Examining these social and logistical factors together call attention to research and tools that warrant greater attention and expansion. Skeels et al. discussed how shared calendars, that allow a patient’s network to organize tasks and responsibilities amongst themselves, can alleviate some of the burden placed on the patient, allowing family and friends to offer help (12). Lotsahelpinghands.com uses many similar ideas, allowing for coordination and communication among patients’ friends and family members. By allowing a patient’s support network to initiate this help, technology removes the responsibility currently placed on the patient to ask for assistance. Further, the technology allows the broader network to ensure continuous social support is available to the patient and that negative changes in support, as described by our participants, do not occur.

The framework offers insight into additional tasks and activities to include in these social-logistical systems. Such tasks include preparing for treatments, help with finances, and dealing with unexpected complications. Allowing one’s support network to become more directly involved in these often overlooked tasks may help patients more effectively manage their health and daily responsibilities. For instance, shared calendars may be used not only to sign up to help with meals and transportation, but may allow family and friends to help with financial management or be “on-call” in case of an unexpected emergency.

Further, an opportunity exists to incorporate the positive aspects of the cancer journey into these tools, such as encouraging the celebration of health and personal milestones. Systems that combine these existing and novel features will allow patients and their care network to address both social and logistical issues, diminishing many obstacles that can derail successful treatment.

### 5.3. Designing across the cancer journey

The cancer journey framework also helps to motivate the design of tools that support patients’ dynamic needs as they move through multiple phases of care. Our participants faced continuous challenges as they transitioned through their care. Many participants struggled with understanding treatment options, preparing for treatments, and managing side effects. They needed support that reached across different phases of care, allowing them to cope with existing challenges as well as prepare for future healthcare changes, which bring new uncertainties.

Our framework calls attention to the value of flexible, adaptive platforms, helping connect patients with personalized sets of tools targeted to their individual and changing experiences. Platforms that curate and promote programs and applications based on patients’ personal information and health records can be used to customize health tools to one’s specific cancer journey. Such tools could help patients, providers, or caregivers ensure that priority needs are covered at each phase of care. For example, providers could find and add financial management tools for newly diagnosed patients with lower socioeconomic status. Resources targeted for survivors after treatment could then surface as patients reach the end of acute care and treatment.

Of course, such health-focused platforms must also consider the personal implications of a cancer diagnosis. We heard repeatedly from the people in our study that they refused to be defined by their cancer diagnosis and many expressed specific strategies in reaffirming existing activities or finding new activities that reflected their true identity. A nuanced design challenge is how to create tools that recognize that cancer invades not just the body, but also many facets of daily life, without redefining all of life as a cancer journey. A possible, yet whimsical, opportunity is a master switch that “turns cancer off” from the computing device when patients need a break and reintegrates cancer information when desired. Such a switch could allow a patient to check their email and calendar without being inundated with cancer-related information.

### 5.4. Evaluation Criteria

We developed the cancer journey framework to offer guidance into designing personalized, holistic tools for cancer patients. To aid in the utilization of this framework across multiple disciplines, we provide a set of heuristics to guide subsequent health IT systems and approaches. These heuristics offer a summative collection of insights to utilize in future IT evaluations. Much like Nielsen’s original usability heuristics, our heuristics should be considered as a broad set of common sense rules than specific usability guidelines (23). We envision that the following ten heuristics will be useful when evaluating personal health technologies designed to support patient-centered care:

- Customize support based on treatment plan
- Customize support based on psychosocial factors and logistic barriers to care
- Customize support based on personal health history and continue support throughout survivorship
- Support coordination and awareness for the care network, including family and informal caregivers
- Provide usable privacy controls
- Anticipate and support challenges (clinical and nonclinical) that emerge during the journey
- Facilitate goal setting and celebrate milestones
- Enable recording and communication of side effects and other patient-centered concerns (e.g. social isolation, patient satisfaction)
- Support patient-centered information management tasks
- Support information seeking that highlights, but it not limited to, provider supplied information

This formative set of heuristics for personalized, holistic technologies offers a summary of lessons learned through our years working with cancer patients. Further research should continue to refine these recommendations. A need exists in the health informatics community to integrate personalized technologies into larger care processes. These heuristics can help those who are not trained in HCI, and who may not be well equipped to assimilate this knowledge in the context of evaluating holistic designs.

## 6. Limitations

One significant limitation from this work is the omission of cancer journeys that incorporate planning for hospice or end of life care. All participants were diagnosed with non-terminal breast cancer. Future work should examine how the cancer journey differs for those with terminal cancer. While our research is also limited by the focus on breast cancer patients, the high degree of similarities between our work and the results found in the literature examining multiple diseases indicates the potential generalizability of our findings towards other cancers and chronic illnesses.

## 7. Conclusion

Patients do not yet possess adequate assistance to manage the full range of needs that arise during and after cancer treatment. All of our participants discussed facing a number of challenges that could significantly impede the success of their treatments. Understanding the range of factors that contribute to a patient’s healthcare experience presents a critical challenge in developing holistic patient tool. To alleviate this challenge, we present a cancer journey framework depicting the significant influences on patients’ cancer journeys. The framework presents designers and researchers with a robust set of patient information that may be critical to consider when developing health tools.

The cancer journey is undoubtedly an overwhelming experience. The emotions, responsibilities, and changes that define the journey can make the experience seem insurmountable. As a community, we must fully understand the make up of these experiences in order to create tools that turn a cancer journey into a more manageable process.

## Footnotes

† 2015. *Global Cancer Facts & Figures 3rd Edition*. American Cancer Society.

‡ Decision Memorandum and Revised Scope of Benefit National Coverage Determination for Speech Generating Devices.
